# Unraveling meteorologically influenced recurrence and sequential infection risks among influenza, mumps, scarlet fever, and hand-foot-and-mouth disease: evidence from Shenzhen, China

**DOI:** 10.3389/fpubh.2026.1920316

**Published:** 2026-08-27

**Authors:** Hongxin Lyu, Huawei Xiong, Jue Liao, Lifang Cai, Yanpeng Cheng

**Affiliations:** 1 Shenzhen Longhua District Center for Disease Control and Prevention (Shenzhen Longhua District Health Supervision Office), Shenzhen, China; 2 Shenzhen Center for Disease Control and Prevention, Shenzhen, China; 3 Academy of Medical Sciences, Shanxi Medical University, Jinzhong, China; 4 Futian District Center for Disease Control and Prevention, Shenzhen, China

**Keywords:** distributed lag nonlinear model, infectious diseases, meteorological factors, recurrence, sequential infection, temperature

## Abstract

**Background:**

Meteorological factors are key triggers for infectious diseases in high-density cities, yet their influence on the acute disease patterns of influenza, mumps, scarlet fever, and hand-foot-and-mouth disease (HFMD) remains unclear. This study aims to reveal the variations in relative risk and secondary infection associated with meteorological factors, thereby providing clues for further confirmatory research.

**Methods:**

We analyzed city-wide incidence data from 2012 to 2020 alongside daily meteorological observations. Distributed lag non-linear models (DLNM) were used to quantify exposure-lag-response relationships. Andersen-Gill models assessed effects on disease recurrence, and Cox models evaluated risks of sequential infections between disease pairs, stratified by season.

**Results:**

The peak-risk temperatures were 14.5 °C for influenza, 18.5 °C for mumps, 17 °C for scarlet fever, and 28.5 °C for hand-foot-and-mouth disease (HFMD). Average temperature (AT) increase raised influenza recurrence risk (e.g., 1.3% higher risk per 1 °C in children aged 0–14 years). Greater temperature variability reduced sequential infection risk for influenza after HFMD in the cold season (Hazard Ratio [HR] = 0.16) and warm season (HR = 0.14). Increased diurnal temperature range also lowered this risk in the cold season (HR = 0.09). Higher AT in warm season reduced sequential infection risks from influenza to HFMD (HR = 0.61). Increased relative humidity was protective in the cold season but became a risk factor in the warm season in specific disease.

**Conclusion:**

Meteorological factors are pivotal in modulating the dynamics of common infections, and influencing both recurrence and sequential transmission. The protective effect of increased temperature variability against secondary infections highlights a complex climate-susceptibility interaction at the population level.

## 1 Introduction

Global climate change and more frequent extreme weather events have drawn widespread concern over their effects on environmental quality and population health (1). The World Health Organization’s COP29 Special Report on Climate Change and Health released in November 2024 noted that climate change will directly induce or worsen the burden of infectious diseases and that cities are critical arenas for climate-health win-win outcomes (2).

Against this backdrop, Asia stands out as a particularly vulnerable epicenter, given its dense populations and diverse climatic zones. Indeed, Asia serves as a central hub for multiple infectious diseases, including influenza, mumps, scarlet fever, and hand, foot, and mouth disease (HFMD). In this context, China’s disease transmission patterns play a pivotal role (3, 4). A national 11-year study of 507 Chinese cities showed that infectious diseases in children and adolescents are largely influenced by cold and hot temperature changes (5).

Building on these national findings, Shenzhen as a densely populated subtropical metropolis offers a compelling case study for how local climate exacerbates these multi-disease threats. The city faces persistent challenges from all four diseases. Influenza circulates with distinct biannual seasonal peaks (6). Mumps remains highly endemic with 16,997 cases documented over a three-year period and scarlet fever has resurged with notable upward trends (7, 8). And HFMD continues to impose a severe burden, with attack rates more than doubling (9).

These alarming local statistics are not merely coincidental. They are intrinsically linked to the city’s unique meteorological conditions, reinforcing the WHO’s emphasis on urban areas as critical intervention points. The subtropical monsoon climate area like Shenzhen makes those diseases the top public health priorities. It not only exhibit significant sex- and age-specific trends but also display predictable seasonal patterns (10). Multiple studies have shown that these variations are largely driven by meteorological factors such as mean temperature, diurnal temperature range, and humidity (11, 12). Previous research has also indicated that both high and low temperatures can increase the risk of respiratory, gastrointestinal, and vector-borne diseases (5).

Meteorological factors affect infectious disease incidence and transmission through dynamic processes that determine seasonal recurrence and inter-disease interactions. Weather conditions directly modulate pathogen transmission efficiency in the environment (13). Indirectly, they also shape population exposure risks by altering human activity patterns, such as indoor crowding and ventilation habits (14). Temperature variability is a primary driver of epidemic peaks, influencing both the timing and amplitude of disease outbreaks (5). Beyond temperature, humidity affects influenza transmission in subtropical areas like Shenzhen by altering aerosol properties and facilitating indoor spread (14). In addition, the intensity of extreme rainfall can significantly affect the increase in diarrhea cases during certain specific months (15). Furthermore, extremely high precipitation, low wind speed, and low sunshine duration increased HFMD reinfection risk in Hefei, China (16). Currently, research on the impact of climatic factors on inter-disease interactions is limited to natural focal and vector-borne diseases (NVBDs), whereas such effects remain largely unreported for common acute infectious diseases (17).

We aimed to construct a comprehensive analytical framework to examine the effects of meteorological factors on four common infectious diseases in Shenzhen. Given the nonlinear and lagged nature of the relationships between meteorological factors and disease incidence, Gasparrini developed the distributed lag nonlinear model (DLNM), which employs time splines to control for seasonality and long-term trends (18). This model overcomes major limitations of traditional time-series approaches in accounting for lag effects, nonlinear associations, and interactions among exposures. In infectious disease research, Cox proportional hazards models are commonly used to analyze the time interval from initial infection to recurrence or reinfection and to identify risk factors for disease recurrence. For instance, a 16-year retrospective cohort study on hand, foot, and mouth disease reinfection applied the Cox model and found that children under 3 years of age, scattered-living children, and urban residence were significant risk factors (19). Additionally, Andersen and Gill proposed the Andersen-Gill counting process model for analyzing recurrent event data of the same disease (20).

Studies have highlighted the occurrence of pathogen co-infections, rendering sequential analysis between infectious diseases feasible (21). Although the seasonal dynamics of infectious diseases are well recognized, a systematic assessment remains lacking regarding the specific response patterns of common infections to meteorological conditions in high-density urban settings, the influence of weather factors on disease recurrence, and their role in triggering sequential infections among different diseases. We aim to provide new evidence for understanding meteorologically driven infectious disease occurrence.

## 2 Methods

### 2.1 Study design and case inclusion

Based on 9 years of surveillance data from 2012 to 2020, this observational environmental epidemiological study first identifies temperature niches for four common acute infectious diseases via DLNM models. Subsequently, Andersen-Gill and Cox models are applied to determine their respective recurrence risks and inter-disease transmission risks. We included all cases clinically or laboratorily diagnosed as influenza, mumps, scarlet fever, and HFMD, reported through the National Notifiable Infectious Disease Reporting System (NNIDRS), with complete onset date and key demographic information.

### 2.2 Data collection

The daily meteorological observations including average temperature (AT), temperature variation (TV), diurnal temperature range (DTR), relative humidity (RH), rainfall (RF), wind speed (WS), and sunshine duration (SD) from the Shenzhen Meteorological Station (ID: 59493). Recurrence was defined as a confirmed case of the same disease reported for the same patient in the NNIDRS at least a longest incubation period after the previous onset. Sequential infection was defined as two different disease diagnoses reported for the same patient in chronological order with at least 14 days between onset dates and without any other study-included disease reported during that interval. This study used the unique personal identifier to sort all reports of the same patient by onset date and construct individual-level disease event sequences.

### 2.3 Modeling

This study utilized Spearman rank correlation analysis and variance inflation factor (VIF) assessment to examine inter-variable associations and diagnose multicollinearity, with the Quasi Akaike Information Criterion (QAIC) applied for model parameter selection. Additionally, sensitivity analyses were conducted by varying the lag period specifications and by including or excluding autocorrelation control, to evaluate the robustness of the model-derived conclusions. Atmospheric pressure was excluded from the DLNM owing to its strong negative correlation with temperature (*r* = −0.854) and comparatively higher collinearity (VIF = 4.54). The DLNM is as follows:

$$\begin{array}{l} \log[E(Y_{t})]=\alpha +\mathrm{cb}({\text{Temp}}_{t},\mathrm{lag})+\sum \mathrm{ns}({\text{Weather}}_{t},df=b)\\ +\mathrm{ns}({\text{Time}}_{t},df=\frac{c}{\text{year}})+\mathrm{as}\cdot \text{factor}({\mathrm{Dow}}_{t})\\ +\mathrm{as}\cdot \text{factor}({\text{Holiday}}_{t})+\beta {\mathrm{res}}_{t-1} \end{array}$$ (1)

*Y_t_* represents the number of cases on day *t*. *α*, *cb*, and *ns* represents the intercept term, the cross basis function and the natural cubic spline function. Temp*_t_*, lag, and Weather*_t_*, respectively, represent average temperature, maximum lag time, and other meteorological factors on day *t*. Time*_t_* represents the time series of day *t*. d*f* is the degree of freedom, and initial degrees of freedom for non-temperature meteorological factors and time are set to *b* and *c*/year, respectively. Dow*_t_* is a multiclass variable indicating whether day *t* falls on Monday to Sunday. Holiday is a binary variable representing Chinese national statutory holidays and vacations for school-age children. res_*t*−1_ represents the first-order lag term of model residuals with corresponding coefficient *β*. Core lag periods were set at 21, 30, 14, and 21 days for influenza, mumps, scarlet fever, and HFMD, respectively, based on their incubation and cumulative effect characteristics. We analyze the lagged risks associated with low (5th percentile) and high (95th percentile) temperatures across sex and age groups.

The Andersen-Gill recurrent event model and Cox proportional hazards model is as follows:

$$h(t|X_{i})=h_{0}(t)\cdot \exp(\beta^{⊤}X_{i})$$ (2)

$\;\;\;h_{i}(t)$ is the hazard function for subject *i* at time *t*. It represents the instantaneous probability of an event occurring at time *t*, given survival up to that time. $h_{0}(t)$ is the baseline hazard function. This is an arbitrary, unspecified, non-negative function of time. It represents the hazard for an individual whose covariates are all zero (or at the reference level). $X_{i}$ is a vector of covariates for subject *i*. β is a vector of regression coefficients to be estimated. It quantifies the log-hazard effect of the covariates. $\exp(\beta^{⊤}X_{i}(t))$ is the hazard ratio (HR). The recurrent event model stratified by age and sex was used to assess the effects of AT, RH, RF, WS and SD on disease recurrence. The sequential infection risks during the cold (November–March) and warm (April–October) seasons were evaluated using predictors including AT, TV, DTR, and RH. Analyses of sequential episodes were restricted to a one-year interval to minimize confounding from environmental changes. Proportional hazards assumptions were verified via Schoenfeld residual tests, and martingale residual plots assessed goodness of fit.

Results are presented as relative risk (RR) in DLNM and hazard ratios (HR) in Andersen-Gill or Cox models, with the 95% confidence interval used to assess statistical risk. All modeling analyses used R version 4.5.0. Detailed parameters are provided in Supplementary material.

## 3 Results

### 3.1 Relative risks brought about by temperature changes

Based on the median temperature of 24.75 °C (with 13.8 °C and 30.0 °C defining the 5th and 95th percentiles, respectively), Supplementary Figures S5–S8 illustrate the satisfactory stability of the DLNM modeling. According to Equation (1), DLNM analyses for the four infectious diseases all revealed inverted U-shaped temperature-risk relationships. It has been proved that all four diseases are affected by the peak temperature risk (Supplementary material). The temperatures of maximum risk were 14.5 °C for influenza, 18.5 °C for mumps, 17 °C for scarlet fever, and 28.5 °C for HFMD. Among the four diseases, influenza has the largest temperature range of risks. Influenza, mumps, and scarlet fever exhibit longer high-risk lag periods under low temperatures, while HFMD does so under high temperatures (Table 1).

**Table 1:** Risk temperature ranges and high-risk lag periods for four infectious diseases.

Diseases	Number of cases	Maximum lag days (d)	Overall risk temperature range (°C)	Maximum risk temperature (°C)	Low-temperature effect (13.8 °C)			High-temperature effect (30.0 °C)		
					High-risk lag day range (d)	Maximum RR (95% CI)	Lag days at max RR (d)	High-risk lag day range (d)	Maximum RR (95% CI)	Lag days at max RR (d)
Influenza	319,303	21	3.5–24.5	14.5	1–21	1.36 (1.24–1.51)	1	2	1.08 (1.03–1.23)	2
Mumps	50,746	30	14.5–24.5	18.5	21–30	1.05 (1.03–1.07)	30	—	—	—
Scarlet fever	16,347	14	9.5–24.5	17	5–11	1.09 (1.05–1.14)	6	1–2	1.27 (1.02–1.58)	1
HFMD	438,541	21	25–31.5	28.5	—	—	—	1–21	1.09 (1.08–1.10)	3

Subgroup analyses showed that influenza risk peaked at 12.5–15 °C, with a predominant low-temperature effect and a lag period of 1 to 21 days across all groups; the highest risk occurred at lag 1 days in the 15–59 y group (RR = 1.53, 95% CI: 1.34, 1.76). Mumps risk was highest at 16–19.5 °C, with significant lag effects only under low temperature over 13 to 30 days, peaking at lag 30 days in the 10–14y group (RR = 1.08, 95% CI: 1.03, 1.12). Scarlet fever peaked at 16-17 °C, driven mainly by low temperature with lags of 5 to 11 days, and was highest in the 0–3 y group (RR = 1.39, 95% CI: 1.01, 1.93). HFMD was primarily high-temperature driven, with lags of 1 to 21 days and early significant risk at lag 2 days in the 1–5 y group (RR = 1.10, 95% CI: 1.08, 1.12) (Supplementary Table S1).

### 3.2 Impact of meteorological factors on disease recurrence

Significant associations were found between meteorological factors and disease recurrence according to Equation (2). Table 2 shows that each additional hour per day of SD increased influenza recurrence risk by 2.6% in those aged 15–39 years (HR = 1.026, 95% CI: 1.002, 1.051) but decreased risk by 6.5% in those aged ≥60 years (HR = 0.935, 95% CI: 0.874, 1.000). Each 1 m/s increase in wind speed reduced influenza recurrence risk by 29.6% in the ≥60 years group (HR = 0.704, 95% CI: 0.559, 0.887) but increased HFMD recurrence risk by 2.7% in children aged 1–5 years (HR = 1.027, 95% CI: 1.009, 1.046). In individuals aged ≥15 years, each 1 mm increase in rainfall was associated with a 0.5% higher risk of mumps recurrence (HR = 1.005, 95% CI: 1.001, 1.010). Higher relative humidity had a weak protective effect in the 1–5 years age group and in males.

**Table 2:** Summary of significant meteorological factors associated with 1 year recurrence of four infectious diseases in Shenzhen.

Diseases	Subgroup	Meteorological factor	HR (95% CI) per 1 − SD increase	*p*-value	Original unit HR (95% CI)
Influenza	15–39 yrs	Sunshine duration	1.102 (1.011–1.201)	0.027	1.026 (1.002–1.051)
	≥60 yrs	Wind speed	0.785 (0.645–0.956)	0.017	0.704 (0.559–0.887)
	≥60 yrs	Sunshine duration	0.773 (0.614–0.975)	0.029	0.935 (0.874–1.000)
Mumps	≥15 yrs	Rainfall	1.110 (1.017–1.211)	0.022	1.005 (1.001–1.010)
HFMD	1–5 yrs	Relative humidity	0.979 (0.960–0.998)	0.030	0.998 (0.996–1.000)
	1–5 yrs	Wind speed	1.022 (1.007–1.038)	0.004	1.027 (1.009–1.046)
	Male	Relative humidity	0.976 (0.954–0.999)	0.044	0.998 (0.995–1.000)
	Male	Wind speed	1.021 (1.003–1.040)	0.023	1.026 (1.004–1.048)
Scarlet fever	All groups	No significant factors	—	—	

One units for meteorological factors: temperature (°C), relative humidity (%), rainfall (mm), wind speed (m/s), and sunshine duration (hours). Hazard ratio (HR) keeps three decimal places to prevent loss of information in this table. Only items with *p* < 0.05 are presented in the table due to numerous meteorological factors.

### 3.3 Effects of temperature and humidity changes on secondary infections

Among 12 pairs of secondary infectious disease cases, 6 were associated with meteorological factors (Table 3). Greater TV was significantly linked to a reduced risk of subsequent infection in specific disease–season settings. In the warm season, the bidirectional risks between HFMD and scarlet fever were both lower. In the cold season, the risk of HFMD (HR = 0.49, 95% CI: 0.30, 0.79) and scarlet fever (HR = 0.31, 95% CI: 0.20, 0.48) after influenza was also reduced. A larger diurnal temperature range reduced the risk of HFMD after influenza in the cold season (HR = 0.40, 95% CI: 0.20, 0.78) but increased the risk of HFMD after scarlet fever in the warm season (HR = 1.98, 95% CI: 1.14, 3.45).

**Table 3:** Risk associations of key meteorological factors with sequential infections in Shenzhen, by season and disease pair.

Diseases	Season	Case count	Average interval (d)	Average temperature (HR, 95% CI)	Diurnal temperature range (HR, 95% CI)	Temperature variability (HR, 95% CI)	Relative humidity (HR, 95% CI)
HFMD → scarlet fever	Warm	68	78.8	0.77 (0.65–0.91)**	0.46 (0.20–1.09, *p* = 0.078)	0.15 (0.08–0.28)***	—
Influenza → HFMD	Cold	38	42	—	0.40 (0.20–0.78)**	0.49 (0.30–0.79)**	0.86 (0.75–0.99)*
Influenza → mumps	Warm	57	76.3	0.47 (0.31–0.74)**	—	0.13 (0.05–0.33)***	—
Influenza → scarlet fever	Cold	65	20	0.74 (0.55–0.99)*	—	0.31 (0.20–0.48)***	—
Mumps → HFMD	Warm	103	67.9	0.71 (0.61–0.82)***	—	0.20 (0.12–0.32)***	—
Scarlet fever → HFMD	Warm	77	58.2	0.57 (0.47–0.69)***	1.98 (1.14–3.45)*	0.08 (0.04–0.15)***	1.09 (1.02–1.18)*

— indicates HR *p*-value >0.1; *, ** and *** indicate *p* < 0.05, *p* < 0.01 and *p* < 0.001, respectively. The complete results can be seen in Supplementary material.

Table 3 revealed that increased AT reduced the risk of secondary infections during the warm season, specifically for scarlet fever following HFMD (HR = 0.77, 95% CI: 0.65, 0.91), HFMD following mumps (HR = 0.71, 95% CI: 0.61, 0.82), and HFMD following scarlet fever (HR = 0.57, 95% CI: 0.47, 0.69). Elevated AT also decreased the risk of scarlet fever following influenza during the cold season (HR = 0.74, 95% CI: 0.55, 0.99). The effect of increased RH varied by season: it was protective against HFMD following influenza in the cold season (HR = 0.86, 95% CI: 0.75–0.99), whereas it emerged as a risk factor for HFMD following scarlet fever in the warm season (HR = 1.09, 95% CI: 1.02, 1.18).

## 4 Discussion

Overall, meteorological factors act as key modulators for the dynamics of four common infectious diseases in Shenzhen. These infectious diseases each exhibit peak risk at specific temperature points, highlighting the existence of pathogen-specific “temperature niches” for infection risk. More importantly, beyond their independent effects, specific meteorological conditions also influence the recurrence and sequential transmission of these diseases. In particular, increased TV and DTR are significantly associated with a reduced risk of sequential infections between certain disease pairs. These protective associations exhibit distinct seasonal patterns, suggesting that climate–susceptibility interactions operate at a systemic level and may influence population susceptibility following prior infections (22).

Outdoor low-temperature conditions likely enhance the survival and spread of respiratory viruses and promote indoor crowding, which increases close contact opportunities (11). This may partly explain the elevated risk of influenza, scarlet fever, and mumps in colder temperature ranges. Influenza and HFMD are also the most sensitive to extreme temperature shifts and show the longest lag effects, indicating strong temperature dependence in their transmission. In Shenzhen, influenza A is the predominant subtype driving seasonal epidemics (23). The immediate risk following sudden temperature drops mainly stems from rapid fluctuations in the diurnal temperature range (24). In contrast, the immediate effect of high temperature on HFMD is largely mediated by specific social contact patterns among children under 5, which sustain ongoing transmission (9, 25).

Our analysis of influenza recurrence identified sunlight duration as a new key factor beyond temperature and humidity. A study conducted in Xiamen found that increased sunlight exposure raises infection risk by promoting physical activity among the population, which may explain why the risk of influenza recurrence in adults increases with longer daylight duration (26). In contrast, the increased influenza risk observed when daily sunlight drops below 2.36 h in Chongqing (27) may be partly attributable to insufficient vitamin D synthesis and compromised immunity, especially among the older adults (28). Furthermore, mumps shows a delayed response to temperature variation, with the peak risk occurring at a 30 day lag. Additionally, it is one of the few acute infectious diseases positively influenced by rainfall, a finding corroborated by studies on the influence of rainfall in China (26).

Additionally, increased outdoor wind speed may reduce the likelihood of outdoor activities among the older adults, thereby exerting a protective effect (29). Nevertheless, a weak but opposite effect on HFMD risk was observed in males and the 1- to 5-year-old group. This could be explained by enhanced viral transmission under higher wind speeds (30). Notably, a Hong Kong study reported a 14- day lagged effect of wind speed on HFMD spread (31). The protective role of rising humidity against HFMD reinfection is a noteworthy finding. Prior studies have demonstrated that low humidity combined with moderate temperatures is associated with an elevated risk of HFMD infection among males (9).

Upon analyzing secondary infections, it was unexpectedly found that prior mumps virus infection attenuates the positive association between warm-season temperature and HFMD incidence. This protective effect is potentially mediated by homocysteine-responsive endoplasmic reticulum protein (HERP), a protein known to activate host innate immunity (32). Contrary to the findings of Lu et al. (33), we observed a protective effect of temperature fluctuations against secondary scarlet fever. As the causative agent of scarlet fever is adapted to a specific temperature range, temperature variation can alter the expression levels of key virulence factors such as pili and hemolysin (34). Similar patterns appear in the seasonal incidence of secondary HFMD, where extreme temperature fluctuations may suppress transmission by limiting pathogen virulence (35).

However, when HFMD occurred secondary to influenza or scarlet fever, DTR and TV exhibited markedly opposite effects. This finding supports the theoretical assertion that the strength or even direction of statistical associations observed at fine temporal scales may dissipate when aggregated over broader temporal scales (36). Furthermore, the secondary effects of humidity on HFMD were not consistent across seasons. This inconsistency may partly stem from pathogen-specific characteristics, but more importantly, it likely reflects the predominant role of the host’s inability to achieve timely thermal adaptation, thereby compromising the maintenance of optimal thermal balance and physiological homeostasis (36).

Future research should involve laboratory studies or integrate population behavior surveys to deeply analyze the specific pathways through which meteorological factors influence disease transmission. Particular focus should be placed on the underlying mechanisms behind the protective effects of temperature variability and average daily temperature range in the context of specific secondary infections. Additionally, comparative studies across multiple cities or regions should be conducted to further refine subgroup classifications and identify high-risk populations.

## 5 Limitations

The interpretation of our findings warrants caution. Given the observational nature of this study, the observed associations reflect correlations rather than causal relationships. Limited age-stratified data for some diseases (e.g., mumps) restrict the generalizability of our conclusions, which warrant further validation. Although we identified strong protective associations between TV, DTR, and recurrent infections, these results should be regarded as exploratory, as we lacked individual-level information on key epidemiological confounders. Moreover, our age-stratified analyses were region-specific and may not generalize to regions with different disease incidence patterns. Therefore, future studies employing longitudinal or experimental designs are warranted to strengthen causal inference.

## Data Availability

The data analyzed in this study is subject to the following licenses/restrictions: the datasets generated and/or analyzed during the current study are not publicly available because they contain patient-related information and their sharing is restricted by local regulations. Data may be obtained from the corresponding author upon reasonable request. Requests to access these datasets should be directed to Hongxin Lyu, 806818334@qq.com.
