# Supplementary material for: Unraveling meteorologically influenced recurrence and sequential infection risks among influenza, mumps, scarlet fever, and hand-foot-and-mouth disease: evidence from Shenzhen, China
Source: Front Public Health. 2026 Aug 27;14:1920316. doi: 10.3389/fpubh.2026.1920316 (PMC13523138; doi:10.3389/fpubh.2026.1920316)
Supplement: Supplementary file 1 [file Supplementary_file_1.docx]

**supplementary materials**

**Methodological exposition**

We used R version 4.5.0 for all modeling analyses. The dplyr and tidyr packages were employed for data filtering, cleaning, and organization; the dlnm package was utilized to fit distributed lag nonlinear models; and the survival package was applied to implement Andersen-Gill recurrent event models and Cox proportional hazards models.The detailed formula is as follows:

***Distributed Lag Nonlinear Model (Formula 1)***

*L*og[*E*(*Y_t_*)]=*α+cb(Temp_t_,lag)+∑ns(Weather_t_,df=b)+ns(Time_t_,df=c/year)+as.factor(Dow_t_)+as.factor(Holiday_t_)+βres_t-1_*	(1)

Sensitivity analyses demonstrated that the exposure-response relationships between temperature and the incidence risks of influenza, scarlet fever, hand-foot-and-mouth disease, and mumps remained consistent across different lag configurations and irrespective of autocorrelation adjustment. Altering the lag intervals (e.g., lag7/14/21 or lag21/30/40) did not substantially affect the overall shape, trend, or temperature range corresponding to peak risk in the exposure-response curves for any of the diseases, indicating that the core associations were robust to variations in lag structure. Furthermore, models adjusting for residual autocorrelation produced risk curves that closely overlapped with those from unadjusted models, with no meaningful change in the observed association patterns. Collectively, these findings support the reliability of the temperature-risk relationships identified for the four diseases across multiple sensitivity scenarios.

Final model specifications were as follows: separate DLNMs were developed for influenza, scarlet fever, hand-foot-and-mouth disease, and mumps to evaluate the effects of meteorological exposures. Temperature served as the primary exposure variable, with degrees of freedom for both the lag and exposure-response dimensions ranging between 3 and 4. To account for long-term trends and seasonal variation, smooth functions of time were incorporated with 2, 2, 7, and 6 degrees of freedom per year for the four diseases, respectively. Other meteorological covariates were uniformly modeled with 3 degrees of freedom to maintain a balance between model flexibility and overfitting risk.

***Andersen-Gill recurrent event models (Formula 2)***

$\begin{matrix} \lambda_{i}(t\mid{\mathcal{\mathcal{F}}}_{t})=\lambda_{0}(t)\exp\left( \beta^{\top}Z_{i}(t) \right) \end{matrix}$	(2)

This study employed a Andersen-Gill recurrent event models to analyze the impact of meteorological factors on the risk of diseases recurrence. The model included five meteorological variables: temperature (TM), relative humidity (RH), rainfall (RF), wind speed (WS), and sunshine duration (SD). All continuous variables were standardized (mean= 0, standard deviation= 1) to eliminate scale effects and facilitate comparison of their effects. The time variable was defined as the interval in days from the first onset to subsequent recurrences, with the endpoint event being disease recurrence. The model used cluster-robust standard errors to adjust for correlations among multiple events within the same patient. The proportional hazards assumption was verified using Schoenfeld residual tests, and residual plots were used to assess model goodness-of-fit.

***Cox proportional hazards models (Formula 3)***

$\begin{matrix} h(t\mid X_{i})=h_{0}(t)\cdot\exp\left( \beta^{\top}X_{i} \right) \end{matrix}$	(3)

Cox proportional hazards models were extended to evaluate the impact of meteorological factors on influenza recurrence risk, employing an Anderson-Gill recurrent events framework to account for multiple infections within the same individual. The analysis was stratified by cold (October–March) and warm (April–September) seasons to capture differential climate–health associations. Predictors included average temperature (℃), diurnal temperature range (maximum–minimum temperature, ℃), temperature variability (standard deviation of daily temperatures over a seven-day moving window), and relative humidity (%). All continuous meteorological variables were standardized (z-scores) to facilitate comparison of effect sizes. The time scale was defined as days from the initial infection to each subsequent recurrence, with robust standard errors clustered by patient to address within-subject correlation. Model assumptions were verified through Schoenfeld residual tests and martingale residual plots. Results are expressed as hazard ratios (HR) with 95% confidence intervals (CI), where HR>1 indicates elevated recurrence risk and HR<1 denotes protective effects.

**Supplementary results**

**A**

**B**

**Figure S1. Overall risk of Influenza**

**A:** The temperature‑related risk of influenza was consistent across sexes, with the largest risk variation observed in the 5–14 age group and the lowest risk in the 0–4 age group.

**B:** At high temperatures, females showed a more pronounced lagged risk than males, though this effect lasted no more than 5 days. A cold‑effect lagged risk was present across all age groups, with the 40-59 age population exhibiting greater risk changes during the initial temperature drop.

**A**

**B**

**Figure S2. Overall risk of Mumps**

**A:** The temperature‑related risk of mumps was similar between sexes. The 10–14 age group showed the greatest risk variation with temperature, with the risk‑prone temperature range concentrated at 15 °C to 20 °C. No significant temperature‑associated risk was observed in individuals aged >14 years.

**B:** No marked sex differences in risk were detected. The 0–14 age group exhibited an elevated risk under prolonged low temperatures, while high temperatures were not associated with a significant risk across all age groups.

**A**

**B**

**Figure S3. Overall risk of Scarlet fever**

A: The temperature‑related risk of scarlet fever was consistent across sexes, with children aged 4–6 years being the primary at‑risk group. Risk began to increase when temperatures exceeded 10 °C and became non‑significant above 25 °C.

B: The cold effect on scarlet fever did not emerge until lag day 3. The 0–3 age group was more sensitive to sustained cold exposure, though the difference from other age groups was modest. High temperatures posed no significant risk across all age groups.

**A**

**B**

**Figure S4. Overall risk of HFMD**

**A:** The global risk temperature range for HFMD was 25°C–32°C. Risk curves were nearly identical across sexes, with higher relative risk extremes observed in the >20 years age group.

**B:** In the 1–5 years age group, risk increased markedly with prolonged high temperatures, and significant risk emerged earliest in this group, peaking at a lag of 2 days.

**Table S1** Risk temperature ranges and high-risk lag periods of sex and age groups for four common infectious diseases in Shenzhen

| Diseases | Groups | Number of cases | Overall risk temperature range (℃) | Maximum risk temperature (℃) | Low-temperature effect (13.8℃) | | | High-temperature effect (30.0℃) | | |
| --- | --- | --- | --- | --- | --- | --- | --- | --- | --- | --- |
|  |  |  |  |  | High-Risk Lag Day Range (d) | Maximum RR(95%CI) | **Lag days at max RR (d)** | High-Risk Lag Day Range(d) | Maximum RR (95%CI) | **Lag days at max RR (d)** |
| Influenza | Male | 173894 | 3.5-24.5 | 14.5 | 1-21 | 1.35(1.21-1.49) | 1 | 2 | 1.07(1.02-1.12) | 2 |
|  | Female | 145409 | 3.5-24.5 | 14.5 | 1-21 | 1.38(1.25-1.53) | 1 | 2-3 | 1.09(1.04-1.14) | 2 |
|  | 0-4y | 100709 | 3.5-24.5, 26.5-27 | 13.5 | 1-21 | 1.24(1.11-1.38) | 1 | - | - | - |
|  | 5-14y | 130074 | 5.5-24.5 | 15 | 1-21 | 1.39(1.20-1.61) | 1 | 2 | 1.13(1.05-1.21) | 2 |
|  | 15-59y | 82288 | 3.5-24.5, 25.5-28 | 14 | 1-21 | 1.53(1.34-1.76) | 1 | 2-3 | 1.10(1.05-1.17) | 2 |
|  | ≥60y | 6232 | 3.5-23.5,  25-28.5 | 12.5 | 1-21 | 1.42(1.08-1.86) | 1 | - | - | - |
| Mumps | Male | 30883 | 14.5-24.5 | 18 | 19-30 | 1.05(1.03-1.08) | 30 | - | - | - |
|  | Female | 19863 | 16.5-24.5 | 18.5 | 25-30 | 1.05(1.02-1.07) | 30 | - | - | - |
|  | 0-4y | 10804 | 18-24.5 | 19.5 | 28-30 | 1.04(1.00-1.08) | 30 | - | - | - |
|  | 5-9y | 21461 | 14.5-24.5 | 18 | 19-30 | 1.06(1.03-1.09) | 30 | - | - | - |
|  | 10-14y | 8511 | 11-24.5,  32.5 | 16 | 13-30 | 1.08(1.03-1.12) | 30 | - | - | - |
|  | ≥15y | 9970 | - | - | - | - | - | - | - | - |
| Scarlet fever | Male | 10343 | 9.5-24.5 | 16.5 | 0,  4-12 | 1.29(1.05-1.59) | 0 | - | - | - |
|  | Female | 6004 | 10.5-24.5 | 17 | 3-11 | 1.10(1.04-1.15) | 5 | - | - | - |
|  | 0-3y | 2948 | 9.5-24.5 | 16 | 0,  4-7, 11-12 | 1.39(1.01-1.93) | 0 | 1-2 | 1.25(1.04-1.52) | 1 |
|  | 4-6y | 10057 | 10-24.5 | 17 | 3-13 | 1.09(1.05-1.14) | 5 | - | - | - |
|  | ≥7y | 3341 | 10.5-24.5 | 16.5 | 3, 5-11 | 1.09(1.05-1.14) | 6 | 1-2 | 1.27(1.02-1.58) | 1 |
| HFMD | Male | 263608 | 25-31.5 | 28.5 | 1-2 | 1.08(1.04-1.12) | 2 | 1-21 | 1.09(1.08-1.10) | 3 |
|  | Female | 174933 | 25-31.5 | 28.5 | 1-2 | 1.08(1.03-1.12) | 2 | 1-21 | 1.09(1.08-1.10) | 3 |
|  | 0y | 66114 | 25-31.5 | 28.5 | 1-2 | 1.08(1.01-1.12) | 2 | 0-21 | 1.09(1.08-1.10) | 4 |
|  | 1-5y | 346336 | 25-31.5 | 28.5 | 1-2 | 1.08(1.04-1.12) | 2 | 1-21 | 1.10(1.08-1.12) | 2 |
|  | 6-20y | 22256 | 25-31 | 28.5 | - | - | - | 3-21 | 1.08(1.05-1.10) | 5 |
|  | ≥21y | 3835 | 25-32 | 29 | - | - | - | 1, 3-18 | 1.11(1.08-1.14) | 9 |

The lag effect was analyzed with P5 temperature as the low temperature (13.8℃) and P95 temperature as the high temperature (30℃). The maximum lag days were the same as overall analysis.

**Table S2** Risk associations of key meteorological factors with sequential infections in Shenzhen, by season and disease pair

| Diseases | Season | Case Count | Average Interval (d) | Average Temperature (HR, 95%CI) | Diurnal temperature range (HR, 95%CI) | Temperature Variability (HR, 95%CI) | Relative Humidity (HR, 95%CI) |
| --- | --- | --- | --- | --- | --- | --- | --- |
| HFMD → Influenza | Cold | 228 | 59 | *NA* | *NA* | *NA* | *NA* |
|  | Warm | 679 | 60.9 | *NA* | *NA* | *NA* | *NA* |
| HFMD → Mumps | Cold | 12 | 59.8 | *NA* | *NA* | *NA* | *NA* |
|  | Warm | 115 | 76.6 | *NA* | *NA* | *NA* | *NA* |
| HFMD → Scarlet Fever | Cold | 19 | 52.3 | *NA* | *NA* | *NA* | *NA* |
|  | Warm | 68 | 78.8 | 0.77 (0.65–0.91)** | 0.46 (0.20–1.09, p=0.078) | 0.15 (0.08–0.28)*** | - |
| Influenza → HFMD | Cold | 38 | 42 | - | 0.40 (0.20–0.78)** | 0.49 (0.30–0.79)** | 0.86 (0.75–0.99)* |
|  | Warm | 1227 | 68.1 | *NA* | *NA* | *NA* | *NA* |
| Influenza → Mumps | Cold | 17 | 44.4 | *NA* | *NA* | *NA* | *NA* |
|  | Warm | 57 | 76.3 | 0.47 (0.31–0.74)** | - | 0.13 (0.05–0.33)*** | - |
| Influenza → Scarlet Fever | Cold | 65 | 20 | 0.74 (0.55–0.99)* | - | 0.31 (0.20–0.48)*** | - |
|  | Warm | 19 | 68.8 | *NA* | *NA* | *NA* | *NA* |
| Mumps → Influenza | Cold | 56 | 47.6 | *NA* | *NA* | *NA* | *NA* |
|  | Warm | 49 | 73.6 | *NA* | *NA* | *NA* | *NA* |
| Mumps → HFMD | Cold | 9 | 64.6 | *NA* | *NA* | *NA* | *NA* |
|  | Warm | 103 | 67.9 | 0.71 (0.61–0.82)*** | - | 0.20 (0.12–0.32)*** | - |
| Scarlet Fever → Influenza | Cold | 89 | 39.5 | *NA* | *NA* | *NA* | *NA* |
|  | Warm | 40 | 51.1 | *NA* | *NA* | *NA* | *NA* |
| Scarlet Fever → HFMD | Cold | 6 | 28.2 | *NA* | *NA* | *NA* | *NA* |
|  | Warm | 77 | 58.2 | 0.57 (0.47–0.69)*** | 1.98 (1.14–3.45)* | 0.08 (0.04–0.15)*** | 1.09 (1.02–1.18)* |
| Mumps ↔ Scarlet Fever | Both² | ≤10 | 61-70 | *NA* | *NA* | *NA* | *NA* |

'-' indicates HR p-value > 0.1; 'NA' was deemed insufficient for reliable Cox model fitting in this study.

'*', '**' and '***' indicate p < 0.05, p < 0.01 and p < 0.001, respectively.

**Table S3** Interannual statistical description of the number of cases and meteorological factors

| Disease and Meteorological factors | Indicators | 2012 | 2013 | 2014 | 2015 | 2016 | 2017 | 2018 | 2019 | 2020 |
| --- | --- | --- | --- | --- | --- | --- | --- | --- | --- | --- |
| Influenza | Mean±SD | 1.07±1.73 | 2.44±3.91 | 6.08±7.01 | 7.66±9.91 | 33.04±53.7 | 51.89±84.59 | 122.95±156.58 | 565.36±503.86 | 83.99±266.88 |
|  | Scope | 0 - 13 | 0 - 37 | 0 - 54 | 0 - 49 | 0 - 300 | 0 - 449 | 0 - 718 | 0 - 2554 | 0 - 1778 |
| Mumps | Mean±SD | 24.33±14.7 | 14.59±6.79 | 10.46±4.79 | 9.52±4.38 | 11.24±5.87 | 24.21±10.19 | 19.99±8.14 | 16.1±5.66 | 8.46±5.05 |
|  | Scope | 5 - 89 | 1 - 35 | 1 - 27 | 0 - 27 | 0 - 41 | 3 - 61 | 1 - 47 | 2 - 36 | 0 - 26 |
| Scarlet Fever | Mean±SD | 2.07±1.95 | 2.44±2.23 | 3.92±2.99 | 4.16±3.86 | 4.24±3.67 | 11.26±8.69 | 8.02±7.37 | 5.95±6.41 | 2.7±6.57 |
|  | Scope | 0 - 10 | 0 - 10 | 0 - 16 | 0 - 25 | 0 - 23 | 0 - 37 | 0 - 46 | 0 - 41 | 0 - 49 |
| HFMD | Mean±SD | 83.84±64.04 | 89.14±84.92 | 135.33±129.65 | 160.45±133.93 | 164.2±131.67 | 171.99±183.25 | 173.39±157.98 | 190.04±193.17 | 32.34±51.89 |
|  | Scope | 2 - 315 | 1 - 375 | 2 - 599 | 0 - 581 | 2 - 548 | 3 - 861 | 0 - 677 | 2 - 790 | 0 - 238 |
| Temperature | Mean±SD | 23.08±5.83 | 23.11±5.16 | 23.25±5.94 | 23.95±5.23 | 23.19±5.87 | 23.65±5.18 | 23.4±5.41 | 23.99±4.5 | 23.84±4.96 |
|  | Scope | 5.85 - 32 | 9.78 - 31.2 | 6 - 31.03 | 11.85 - 32.95 | 3.48 - 32.15 | 9.6 - 32.43 | 6.63 - 30.8 | 11.25 - 30.93 | 9.38 - 30.65 |
| Humidity | Mean±SD | 78.72±11.12 | 74.73±15.51 | 72.93±12.73 | 71.82±11.04 | 78.89±11.44 | 76.83±13.35 | 75.81±12.9 | 77.46±12.5 | 76.07±11.43 |
|  | Scope | 31.5 - 100 | 24.25 - 99.5 | 18.5 - 95.75 | 28 - 93 | 29 - 98.5 | 28.5 - 99 | 23 - 97.5 | 28.5 - 97 | 29 - 98.25 |
| Rainfall | Mean±SD | 4.24±12.79 | 5.96±14.23 | 5.14±20.16 | 4.28±15.24 | 7.02±19.58 | 5.46±16.7 | 5.48±15.83 | 5.6±14.88 | 16.79±47.75 |
|  | Scope | 0 - 153 | 0 - 97.1 | 0 - 247 | 0 - 149 | 0 - 166.4 | 0 - 162.1 | 0 - 174 | 0 - 108 | 0 - 352.3 |
| Wind speed | Mean±SD | 2.28±0.75 | 2.17±0.82 | 2.16±0.75 | 1.99±0.67 | 1.85±0.77 | 1.83±0.8 | 1.9±0.76 | 1.88±0.81 | 2.02±0.82 |
|  | Scope | 1 - 6 | 0.25 - 5.5 | 0.5 - 4.5 | 0.67 - 4.75 | 0.75 - 5.25 | 0.5 - 5.75 | 0.75 - 6.5 | 0.5 - 5.75 | 0.75 - 6 |
| Atmospheric pressure | Mean±SD | 758.67±5 | 759.55±4.78 | 759.84±5 | 760.1±4.96 | 759.69±5.33 | 760.05±4.63 | 759.54±5.22 | 759.57±4.92 | 760.16±4.46 |
|  | Scope | 743.00 - 769.50 | 745.53 - 770.23 | 749.53 - 770.83 | 748.38 - 770.30 | 745.60 - 776.58 | 747.33 - 771.15 | 745.30 - 771.33 | 748.00 - 770.85 | 750.25 - 770.90 |
| sunlight | Mean±SD | 4.88 ± 3.67 | 5.24 ± 3.83 | 5.57 ± 3.91 | 5.38 ± 3.71 | 4.81 ± 3.85 | 5.26 ± 3.90 | 5.22 ± 3.85 | 5.12 ± 3.58 | 5.15 ± 3.73 |
|  | Scope | 0 - 12.20 | 0 - 12.50 | 0 - 12.30 | 0 - 12.30 | 0 - 12.20 | 0 - 12.00 | 0 - 12.00 | 0 - 11.70 | 0 - 11.70 |

Statistical descriptions are conducted based on daily data.

**Sensitivity analysis of DLNM**

**We only display the most reliable sensitivity analysis charts.**

**Figure S5 Influenza**

**Figure S6 Mumps**

**Figure S7 Scarlet fever**

**Figure S8 HFMD**

**Figure S9 Combined effects of temperature and lag days on total population influenza incidence**

**Figure S10 Combined effects of temperature and lag days on total population mumps incidence**

**Figure S11 Combined effects of temperature and lag days on total population scarlet fever incidence**

**Figure S12 Combined effects of temperature and lag days on total population HFMD incidence**
